# Metabolic Profiling and In Vitro Assessment of the Immunomodulatory Effects of Hydrodistillation-Derived Extracts from the Fruticose Lichen *Pseudevernia furfuracea* (L.) Zopf. on Human Lymphocytes

**DOI:** 10.3390/jox15060201

**Published:** 2025-12-01

**Authors:** Yasser Essadki, Antonio Casas-Rodríguez, Antonio Cascajosa-Lira, Leticia Diez-Quijada, Alexandre Campos, Vitor Vasconcelos, Fatima El Khalloufi, Brahim Oudra, Ana M. Cameán, Angeles Jos

**Affiliations:** 1 Water Sciences, Microbial Biotechnologies and Sustainability of Natural Resources Laboratory (Aquabiotech), Faculty of Sciences Semlalia of Marrakech, Cadi Ayyad University, UCA, Av. Prince My Abdellah, P.O. Box 2390, Marrakech 40000, Morocco; yasser.essadki@ced.uca.ma (Y.E.); oudra@uca.ac.ma (B.O.); 2 Area of Toxicology, Faculty of Pharmacy, University of Sevilla, C. Tramontana, 2, 41012 Sevilla, Spain; acasasr@us.es (A.C.-R.); aclira@us.es (A.C.-L.); camean@us.es (A.M.C.); angelesjos@us.es (A.J.); 3 Interdisciplinary Centre of Marine and Environmental Research (CIIMAR/CIMAR), University of Porto, Terminal de Cruzeiros do Porto de Leixões, 4450-208 Matosinhos, Portugal; amoclclix@gmail.com (A.C.); vmvascon@fc.up.pt (V.V.); 4 Department of Biology, Faculty of Sciences, University of Porto (FCUP), Rua Campo Alegre s/n, 4169-007 Porto, Portugal; 5 Natural Resources Engineering and Environmental Impacts Team, Multidisciplinary Research and Innovation Laboratory, Polydisciplinary Faculty of Khouribga, Sultan Moulay Slimane University of Beni Mellal, Bd 2 Mars, Khouribga 25000, Morocco; elkhalloufi.f@gmail.com

**Keywords:** lichens, *Pseudevernia furfuracea*, composition, extracts, immunotoxicity, Jurkat cells

## Abstract

Lichens are complex symbiotic systems known for synthesizing diverse secondary metabolites with documented antimicrobial, antioxidant, and antiproliferative activities. The present study focused on *Pseudevernia furfuracea*, a species widely distributed across Moroccan habitats. Two hydrodistillation-derived extracts (HE1 and HE2) were analyzed through ultra-high-Performance liquid chromatography coupled with tandem mass spectrometry (UHPLC-MS/MS) to characterize their metabolite composition, and their effects were evaluated on Jurkat cells, a representative human cell line of the immune system. As the results of the characterization, the main compounds identified were Caprolactam, N,N-Diethylaniline, Erucamide, and 4-Isopropylaniline. Cytotoxicity assessment revealed that both HE1 and HE2 decreased the viability of Jurkat cells in a concentration-dependent manner. The mean effective concentrations (EC_50_) after 24 h of treatment were 53.79 ± 2.92 µg/mL for HE1 and 59.76 ± 2.01 µg/mL for HE2. Cell death mechanisms were further examined by flow cytometry, revealing that apoptosis predominated after 24 h of treatment, progressing mainly to late apoptotic stages after 48 h. In parallel, the expression levels of key cytokine genes, including IL-2, TNF-α, and IFN-γ, were quantified at the mRNA level to evaluate potential immunomodulatory effects. Up-regulation was observed in IL-2 after exposure to both extracts for 24 and 48 h, and in the case of IFN-γ after exposure to HE2 for 24 h; in contrast, HE1 and HE2 produced down-regulation in TNF-α at 24 h. These findings suggest that HE1 and HE2 have immunomodulatory activity in Jurkat cells. Further investigations are needed to elucidate the underlying mechanisms and to clarify how HE1 and HE2 influence immune responses in human systems.

## 1. Introduction

Lichens are symbiotic organisms known for their unique secondary metabolites [1], which contribute to their ecological adaptability [2] and potential pharmacological applications [3]. More than a thousand secondary metabolites originating from lichens have been identified [4], many of which exhibit antioxidant [5], antimicrobial [6], and anticancer properties [7]. To date, research has primarily focused on secondary metabolites such as depsides, depsidones, and dibenzofurans, which can represent up to 15–20% of the lichen dry weight. In contrast, other secondary metabolites—often present in much lower concentrations—remain largely underexplored [8].

In our study, we investigated lichen extracts obtained via hydrodistillation, a method rarely applied in lichen research. Drawing parallels from plant systems, where metabolites such as monoterpenes and phenolics play crucial ecological and signaling roles [9], this suggests that lichens may harbor a broader and more chemically diverse arsenal of bioactive molecules than previously recognized. Moreover, these less-characterized lichen secondary metabolites may also possess promising pharmacological effects.

Although several lichen extracts produced through comparable extraction techniques have been examined for their antioxidant and antimicrobial potential [10], no systematic screening of their cytotoxic or anticancer effects has been performed. Moreover, previous research on lichen bioactivity has largely relied on organic solvent extractions, which yield different classes of metabolites than those obtained via hydrodistillation. Consequently, the potential of novel compounds present in lichen extracts remains an unresolved area of investigation within cancer research.

Extensive research has been devoted to characterizing the chemical profile and assessing the diverse biological activities of *Pseudevernia furfuracea* [11,12,13,14,15,16], although the mechanisms involved in these activities are not fully elucidated. It has been demonstrated that nonpolar fractions of *Pseudevernia furfuracea* have significant antimicrobial activity, whereas polar fractions have been reported to exhibit antioxidant, anti-inflammatory, antinociceptive, and wound-healing potential [11]. Aoussar et al. [13] reported that among three different lichens from Morocco, the extract of *Pseudevernia furfuracea* exhibited the strongest antioxidant activity, contained the highest total phenolic content, and showed the most pronounced antibacterial potential. In addition, extracts from *P. furfuracea* demonstrated cytotoxic effects against several cancer lines [12,16]. The potential antiproliferative and cytotoxic activity of *P. furfuracea* extracts has been also demonstrated in vitro in 2D and 3D models [15]. Moreover, Furmanek et al. [16] reported high cytotoxic activity for extracts from this lichen species in different cancer cell lines.

Building on our prior work investigating the antimicrobial potential of this novel type of extract from lichens [17], we now aim to assess their cytotoxic potential and impact on mRNA expression and types of cell death. In this study, we evaluated the biological effects of two extracts (HE1 and HE2) obtained from distinct Moroccan biotopes on Jurkat cells, a model of human lymphocytes, under the hypothesis that these extracts harbor bioactive metabolites capable of triggering cytotoxic responses through specific molecular mechanisms. Jurkat cells has been widely used in the research of T-cell biology [18]. As an immortalized T-cell line, it provides a reliable in vitro model of adaptive immune responses owing to its phenotypic and functional similarity to human lymphocytes [19]. However, to date, no previous studies have been carried out on these extracts in this cell line.

The present study reports the first in vitro evaluation of the immunomodulatory properties of *Pseudevernia furfuracea* extracts HE1 and HE2 using Jurkat cells as the experimental model. Cytotoxic assessment was performed using the Trypan Blue exclusion technique. The expression levels of cytokine genes (IL-2, TNF-α, and IFN-γ) were analyzed using real-time quantitative polymerase chain reaction (RT-qPCR) to evaluate possible inflammatory responses, and flow cytometry was used to characterize cell death pathways, apoptosis and necrosis, after treatment with the extracts. In addition, this work presents the first UHPLC–MS/MS characterization of the chemical composition of both extracts. The potential transformation products of atraric acid, the predominant constituent, were also studied to gain insight into its contribution to the observed biological effects.

## 2. Materials and Methods

### 2.1. Chemical and Reagents

All cell culture media and reagents were sourced from commercial suppliers. Media components were obtained from Gibco (Biomol, Sevilla, Spain), while chemicals used in cytotoxicity and flow cytometry assays were purchased from Sigma-Aldrich (Madrid, Spain). Primers specific for IL-2 (qHsaCIP0029918), TNF-α (qHsaCEP0040184), and IFN-γ (qHsaCEP0050640), together with the materials required for gene expression analysis, were provided by Bio-Rad Laboratories (Hercules, CA, USA) and Qiagen (Madrid, Spain).

### 2.2. Lichen Sample Collection and Extraction

Samples of *Pseudevernia furfuracea* were collected in June 2021 from two Moroccan regions: the High Atlas near Marrakech and the Middle Atlas near Azrou [17]. Lichen identification was conducted by one of the authors (Y.E.) using determination keys [20] and online resources [21].

Lichen extraction was performed according to the procedure previously described by Essadki et al. [17]. Briefly, 100 g of powdered lichen was subjected to hydrodistillation. The obtained distillate was afterwards extracted with dichloromethane. The dry residues obtained after dichloromethane evaporation were stored in at −20 °C. Extracts from *Pseudevernia furfuracea* collected in both Atlas regions were designated as HE1 and HE2, respectively.

### 2.3. Chemical Characterization

The extracts HE1 and HE2 were previously characterized using GC-MS by Essadki et al. [17] but only with regard to volatile compounds. The concentrations of atraric acid and chloroatranol were 73.53% and 19.80%, respectively, in HE1 and 56.95% and 24.38%, respectively, in HE2, being identified as the main components. To achieve a more comprehensive chemical profile, the present study expands upon this analysis by examining non-volatile compounds through UHPLC-MS/MS.

#### UHPLC-MS/MS Equipment and Conditions

Compound profiling of HE1 and HE2 followed the analytical workflow described by Cascajosa-Lira et al. [22], with minor adaptations. The analyses were performed on a Thermo Scientific UHPLC system (Dionex Ultimate 3000RS) interfaced with a Q Exactive quadrupole–Orbitrap mass spectrometer (Thermo Fisher Scientific, Waltham, MA, USA equipped with a HESI-II ion source. Instrument control and data handling were managed through Xcalibur software (v4.3, Thermo Fisher Scientific).

Chromatographic separation was achieved using an Acquity UPLC BEH C18 column (2.1 × 100 mm, 1.7 µm; Waters, Milford, MA, USA) maintained at a flow rate of 0.4 mL min^−1^. The mobile phase comprised water (A) and methanol (B), each containing 0.1% formic acid. The gradient program was as follows: 0–0.5 min, 5% B; 0.5–5 min, linear increase to 100% B; 5–7 min, hold at 100% B; and 7–10 min, re-equilibration at 5% B, with an injection volume of 5 µL.

Mass detection was conducted in both positive and negative ionization modes under full-scan acquisition at 70,000 resolution (FWHM, *m*/*z* 200). The HESI settings were as follows: spray voltage 3.5 kV (positive) and 3.0 kV (negative), S-lens RF level 50, capillary temperature 320 °C, and probe heater 425 °C. Sheath, auxiliary, and sweep gas flows were adjusted to 50, 13, and 3 arbitrary units, respectively. Data were processed and interpreted using TraceFinder 5.1 and Compound Discoverer 3.2 (Thermo Fisher Scientific).

### 2.4. Cell System

The human T-lymphoblastic Jurkat cell line (ATCC^®^ TIB-152), derived from a case of acute T-cell leukemia, was sourced from the American Type Culture Collection (ATCC, Manassas, VA, USA). Cells were cultured at 37 °C in a humidified atmosphere containing 5% CO_2_ at the Biology Service of the Centro de Investigación, Tecnología e Innovación, Universidad de Sevilla (CITIUS). Growth was supported in RPMI-1640 medium (R8005) supplemented with 10% fetal bovine serum, 2 g L^−1^ sodium bicarbonate, 10,000 U mL^−1^ penicillin, and 10 mg mL^−1^ streptomycin. Standard aseptic techniques were applied throughout, and all experiments were performed with cells between passages 5 and 18.

### 2.5. Cytotoxicity Evaluation

Cell viability was determined according to the Trypan Blue exclusion assay described by Strober [23]. Jurkat cells were plated in 96-well microplates at a density of 5 × 10^5^ cells mL^−1^ and allowed to stabilize for 24 h before exposure to graded concentrations of HE1 and HE2. Extract stock solutions (100 mg mL^−1^) were prepared in DMSO, and were serially diluted to yield final treatment concentrations ranging from 20 to 200 µg mL^−1^. Three experimental controls were included: untreated cells (negative control), a solvent control containing 0.2% DMSO, and a positive control treated with 0.3% Triton X-100. As no significant differences were found between the negative and solvent controls, only data from the solvent control are reported. Cell viability was quantified by counting unstained (live) and blue-stained (non-viable) cells in a Neubauer chamber, and results are expressed as the percentage of viable cells. Each assay was independently repeated at least three times, with all concentrations tested in triplicate.

### 2.6. Flow Cytometry

Flow cytometry was conducted according to the approach of Casas-Rodríguez et al. [24]. Jurkat cells (1 × 10^6^ cells well^−1^) were seeded in six-well plates and exposed for 24 h or 48 h to different concentrations of HE1 and HE2. The exposure levels corresponded to each extract’s EC_50_ value and its respective half and quarter dilutions. Experimental controls comprised untreated cells (negative control), cells maintained in medium with 0.2% DMSO (solvent control), and a positive control treated with 1.5 µM camptothecin.

After treatment, cells were collected by centrifugation, rinsed with PBS, and resuspended in 500 µL of annexin-binding buffer. Staining was carried out by incubating the suspensions for 15 min at room temperature with 5 µL Annexin V-FITC (apoptotis marker) and 1 µL propidium iodide (necrosis marker). Subsequently, 400 µL of binding buffer was added, and fluorescence signals were measured on a MACSQuant VYB cytometer (Miltenyi Biotec, Bergisch Gladbach, Germany). For each sample, at least 10,000 events were acquired. Because no significant differences were detected between the negative and solvent controls, only solvent control data are reported.

### 2.7. Gene Expression Analysis

Quantitative reverse transcription PCR (RT-qPCR) was employed to assess the mRNA expression of selected cytokines, following the methodology of Casas-Rodríguez et al. [24]. Jurkat cells were treated with non-cytotoxic concentrations of HE1 and HE2 (20 µg mL^−1^) for 24 h and 48 h. Experimental controls comprised untreated cells (negative control), a solvent control containing 0.2% DMSO, and a positive control exposed to 10 ng mL^−1^ lipopolysaccharide (LPS). The GAPDH housekeeping gene was used as an internal control.

Total RNA was extracted using an RNeasy Mini Kit (Qiagen, Hilden, Germany), and any residual genomic DNA was eliminated using an RNase-Free DNase Set (Qiagen). Complementary DNA (cDNA) was synthesized from 1 µg of total RNA with a QuantiTect^®^ Reverse Transcription Kit (Qiagen) and diluted 1:5 before amplification. qPCR reactions were carried out on a LightCycler^®^ 480 system (Roche, Basel, Switzerland) using PrimePCR probes (Bio-Rad Laboratories, Hercules, CA, USA) specific for IL-2 (qHsaCIP0029918), TNF-α (qHsaCEP0040184), and IFN-γ (qHsaCEP0050640). Thermal cycling was performed with an initial denaturation at 95 °C for 2 min, followed by 50 cycles of 95 °C for 5 s and 60 °C for 30 s.

Relative expression levels were determined using the 2^−ΔΔCT^ method. Fold change values below 0.7 were interpreted as gene down-regulation, whereas values above 1.5 indicated up-regulation. Since no statistically significant differences were detected between the negative and solvent controls, only results for the solvent control are presented.

### 2.8. Statistical Analysis

Data distribution and variance homogeneity were assessed using the Kolmogorov–Smirnov test. For datasets meeting normality assumptions, one-way ANOVA followed by Tukey’s multiple comparison test was applied. Non-parametric data were analyzed using the Kruskal–Wallis test with Dunn’s post hoc comparisons. EC_50_ values were estimated by linear regression from the corresponding concentration–response curves. All statistical procedures were performed in GraphPad Prism version 9.0 (GraphPad Software, USA). Differences were considered statistically significant at * p < 0.05, ** p < 0.01, *** p < 0.001, and **** p < 0.0001.

## 3. Results and Discussion

### 3.1. Putative Identification of Compounds by UHPLC-MS/MS

The UHPLC-MS/MS analysis led to the putative identification of 43 compounds in both HE1 and HE2 (Table 1). In the case of HE1, 21 compounds were detected, with 39 for HE2 and a total of 17 shared between HE1 and HE2. Moreover, only 4 compounds were identified specifically in HE1 and 22 were characteristic of HE2. The most abundant compounds in terms of area under the curve were Caprolactam, N, N-Diethylaniline, Erucamide, and 4-Isopropylaniline. The most represented families of compounds were aromatic amines (six compounds), amphetamine-like stimulants and phenethylamines, phthalic derivatives (three compounds), and amides and fatty acids amides (three compounds).

The extracts contain a chemically diverse mixture comprising several major classes. Phenolic compounds, such as substituted naphthalenetriols (1,3,8-Naphthalenetriol) and alkylated phenols (3,5-di-tert-Butyl-4-hydroxybenzaldehyde, Eugenol methyl ether), are present alongside terpenoid derivatives like menthyl acetate ((-)-Menthylacetate) and hygrophorone analogs (4-O-acetyl hygrophorone A14). A significant portion includes fatty acids and their derivatives, including amides and esterified forms (oleamide, stearoyl ethanolamide, Erucamide, dibutyl hexanedioate). Various aromatic and aliphatic amines, particularly aniline derivatives (2,6-Diethylaniline, N,N-Diethylaniline, 4-Isopropylaniline), are also included. Alkaloid-like structures and nitrogen-containing heterocycles are represented (Bacillamidin G, Berkazaphilone A, 2-oxa-4-azatetracyclo [6.3.1.1~6,10~.0~1,5~]tridecan-3-one), along with lactones and lactams (Caprolactam, Thermolide E). Additionally, the extracts contain esters, both aliphatic and aromatic (diethyl phthalate, dibutyl phthalate, 1,2,2,6,6-Pentamethyl-4-piperidinyl acrylate), quinones (9,10-Phenanthraquinone), anhydrides (phthalic anhydride), and several industrial chemicals such as glycols, phosphates, and nitrosamines (triethylene glycol, tributyl phosphate, Di-N-butylnitrosoamine). These compositions highlight a wide range of chemical families of natural product-like structures, especially in the case of HE2.

In previous studies, our group characterized the chemical nature of these extracts based on volatile components [17]. In that study, GC-MS analysis was performed, revealing volatile compounds that closely align with the chemical profile present in this work. The application of both methodologies allow us the identification of both volatile, apolar, thermostable compounds and non-volatile compounds. This dual approach provides a more complete metabolic fingerprint of these extracts, enhancing our understanding of their bioactive potential. Thus, monoterpenes and sesquiterpenes such as trans-verbenol, δ-cadinene, and himachalol detected in the previous work are consistent with the presence of menthyl acetate and other terpenoid derivatives found in the non-volatile fraction. Similarly, volatile phenolic compounds like acetyl isoeugenol support the identification of related aromatic ethers such as eugenol methyl ether in the present work. The detection of saturated and unsaturated fatty acids, including n-hexadecanoic acid and 9,12,15-octadecatrienoic acid, aligns with the presence of amide and ester derivatives of fatty acids in the original profile, such as oleamide and stearoyl ethanolamide. Furthermore, volatile compounds typical of lichen secondary metabolites, such as methyl haematommate, atraric acid, and chloroatranol, further support the authentic lichen origin of the extract [17]. However, due to their volatile nature, these compounds were not detected using UHPLC-MS/MS. Instead, in the next section, we focus on the characterization of the hydrophilic derivatives of atraric acid, detected in the present work by Compound Discoverer.

### 3.2. Putative Identification of the Transformation Products of Atraric Acid

**Figure 1:**
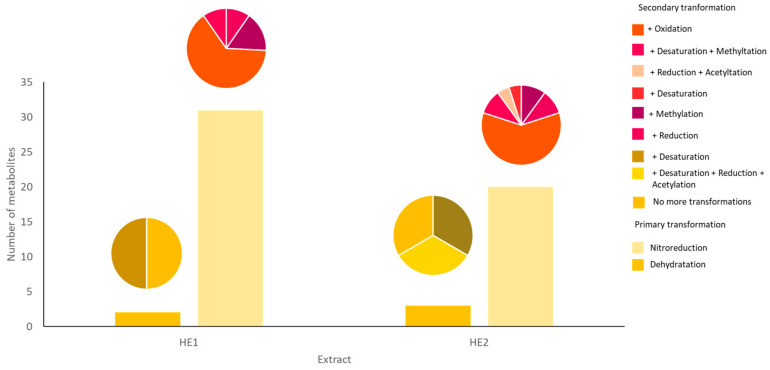
Graphical representation of the putative chemical transformations of atraric acid detected through UHPLC-MS/MS analysis and Compound Discoverer software.

The presence of primary and secondary transformation products of atraric acid was identified (Table 2, Figure 1). The most frequent transformations were mainly nitroreduction followed by dehydration. The secondary transformations were oxidation, desaturation, and methylation. Acetylation was detected in HE2 but not in HE1.

In this study, we focused on the chemical characterization of the secondary metabolites derived from atraric acid, a major component (73.53% in HE1 and 56.95% in HE2) according to Essadki et al. [17] found in *Pseudevernia furfuracea* extracts. Through UHPLC-MS/MS analysis, several hydrophilic derivatives of atraric acid were identified, which further confirm the complex chemical nature of this lichen. While previous works, like those by Güvenç et al. [11], have identified a range of depsidic compounds in the methanolic extract of *P. furfuracea* harvested from Turkey, our study distinguishes itself by focusing on the atraric acid pathway and its hydrophilic derivatives. We did not observe the classic depsidic compounds like protocetraric acid or atranorin in our analysis, which highlights the novelty in terms of composition of our specific extracts obtained by hydrodistillation compared to classical organic solvent extracts. These findings enhance our understanding of the secondary metabolite profile of *Pseudevernia furfuracea* and underscore the diversity of chemical compounds that can be derived from a single metabolic precursor, atraric acid. The identification of these derivatives adds a novel layer to the chemical complexity of this lichen, offering potential insights for further pharmacological studies.

This work represents, to our knowledge, the first identification of atraric acid derivatives in lichen species. Although earlier investigations, including the work of Noël et al. [25], have investigated the biotransformation of other depsidic compounds, specifically usnic acid, resulting in the identification of ethanolanime and methylated derivatives, our findings reveal methylated products that likely follow a similar metabolic pathway, supported by the structural resemblance between the parent compounds. Nevertheless, we cannot exclude the impact of the hydrodistillation procedure on the composition of the extracts. Indeed, it was previously reported that heating in presence of water can elicit hydrolysis and other chemical transformations of various compounds contained in the extracts [26].

### 3.3. Determination of Cell Viability

**Figure 2:**
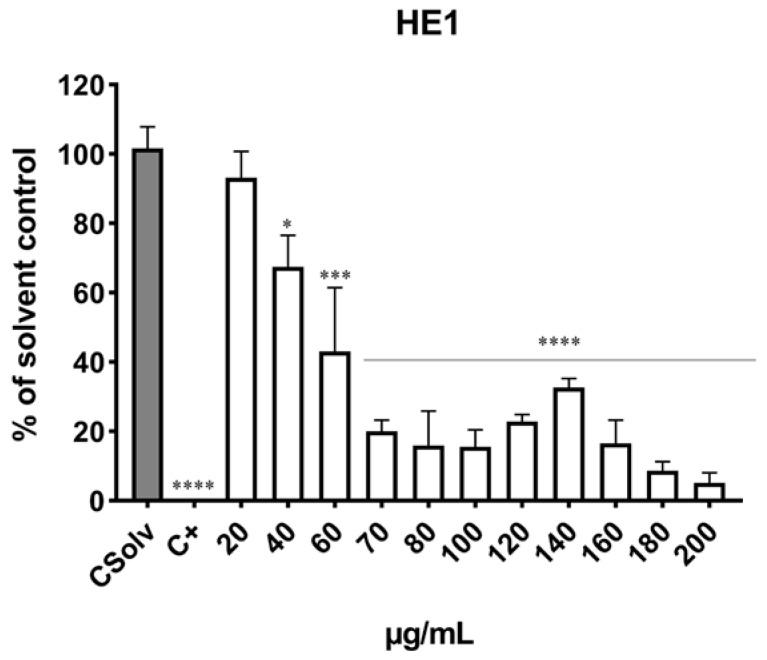
Jurkat cells (5 × 10^5^ cells/mL) were exposed for 24 h to increasing concentrations of HE1. Cell viability was evaluated using the Trypan Blue exclusion assay, which distinguishes viable (unstained) from non-viable (blue-stained) cells under microscopy. Triton X-100 (0.3%) served as a positive control for complete cell lysis, and 0.2% DMSO was used as solvent control. Results are expressed as mean ± SD from three independent experiments performed in triplicate. Statistical significance vs. solvent control: * *p* < 0.05, *** *p* < 0.001, and **** *p* < 0.0001.

**Figure 3:**
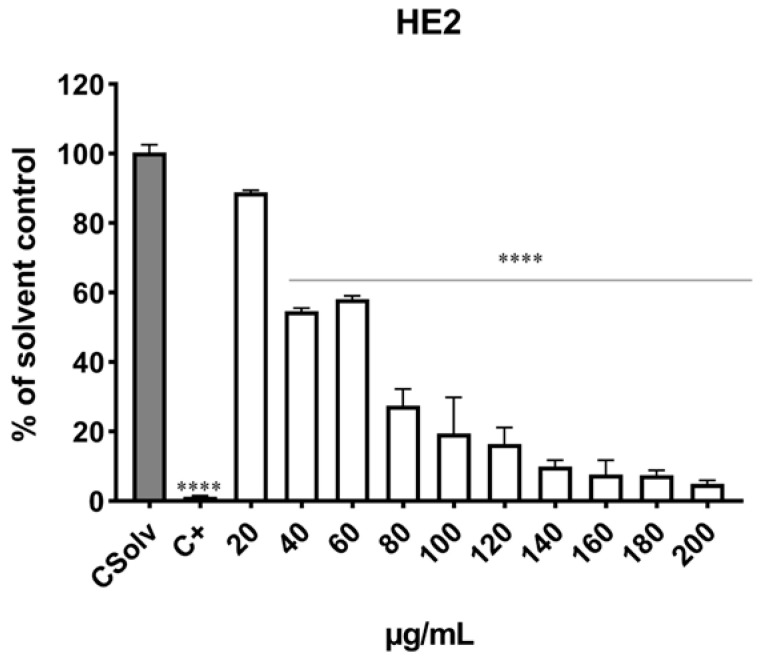
Jurkat cells (5 × 10^5^ cells/mL) were exposed for 24 h to increasing concentrations of HE2. Cell viability was evaluated using the Trypan Blue exclusion assay, which distinguishes viable (unstained) from non-viable (blue-stained) cells under microscopy. Triton X-100 (0.3%) served as a positive control for complete cell lysis, and 0.2% DMSO was used as solvent control. Results are expressed as mean ± SD from three independent experiments performed in triplicate. Statistical significance vs. solvent control: **** *p* < 0.0001.

As shown in Figure 2 and Figure 3, exposure of Jurkat cells to HE1 and HE2 for 24 h resulted in a dose-dependent decline in viability, with statistically significant effects observed from 40 µg mL^−1^ onward compared with the solvent control. For HE1, marked cytotoxicity was observed after exposure to ≥70 µg/mL in which viability was significantly decreased (up to 5.20% viability). For HE2, the highest cytotoxicity was observed after exposure to ≥80 µg/mL with decreases in cell viability (up to 4.94% viability). Positive control (0.3% Triton X-100) produced a reduction in cell viability of more than 90%. The EC_50_ values were 53.79 ± 2.92 µg/mL and 59.76 ± 2.01 µg/mL for HE1 (Figure 2) and HE2 (Figure 3), respectively.

Cytotoxicity testing represents a fundamental step in in vitro toxicity assessment. The present findings demonstrate that HE1 and HE2 extracts from *Pseudevernia furfuracea* var. *furfuracea* significantly reduced Jurkat cell viability, with both extracts exhibiting comparable cytotoxic potential. According to the American National Cancer Institute (NCI) guidelines, crude plant extracts are considered cytotoxic when their IC_50_ values fall below 30 µg mL^−1^ in preliminary screening assays [27]. To our knowledge, studies that have evaluated the cytotoxicity of *Pseudevernia furfuracea* in the same cells are very scarce [15,16,28]. The results obtained here align with the observations previously described by Kello et al. [28]. These authors assessed the cytotoxic effects of an extract from *P. furfuracea* (10, 50, 100 µg/mL) in Jurkat cells using resazurin assay and confirmed its cytotoxic effects, which were concentration- and time-dependent, obtaining an IC_50_ value of 45 µg/mL. Similar results were reported by the same authors [15], who evaluated the cytotoxicity of *P. furfuracea* extracts (10–100 µg/mL) in Jurkat cells using the resazurin assay and obtained an IC_50_ of 44.3 µg/mL in a 2D model. In addition, they compared the results in 2D with those obtained in a 3D spheroids model and reported an IC_50_ of 77.3 µg/mL. Differences in the effects of the same extracts or metabolites between 2D and 3D culture systems could be influenced by factors such as compound diffusion and accessibility to proliferating cells at the surface versus quiescent or necrotic cells in the inner regions, as consequences of oxygen and nutrient transport limitations [15]. In contrast, Furmanek et al. [16] observed reduced cytotoxicity in Jurkat cells, obtaining an IC_50_ of 174.87 µg mL^−1^ after treatment with *Pseudevernia furfuracea* extracts (1–500 µg mL^−1^) evaluated using the MTS method. These differences could be explained in part due to the presence of different compounds and metabolites from these lichen species as a result of the differences in environmental conditions, extraction procedures employed, time of exposure, etc.

There are several studies focused on the cytotoxicity of compounds and/or major metabolites from *P. furfuracea* extracts [12,15,16,29] and different results have been reported depending on their main components. In relation to the compounds present in HE1 and HE2 extracts, the main components were Caprolactam, N, N-Diethylaniline, Erucamide, and 4-Isopropylaniline. In comparison, in the work of Furmanek et al. [16], the major phenolic constituents identified were 3-hydroxyphysodic acid, 2′-O-methylphysodic acid, and a physodic acid isomer. Physodic acid and its derivatives have been reported to contribute to the notable antioxidant and antitumor potential of *Pseudevernia furfuracea* extracts [16]. This interpretation is further supported by the findings of Kello et al. [15], who demonstrated a pronounced cytotoxic activity of physodic acid against Jurkat cells. Given the scarcity of studies addressing the cytotoxic behavior of *P. furfuracea* in this cell line, the present results provide novel and promising evidence of biological activity in this lichen species. Further research is warranted to elucidate the precise mechanisms underlying these effects in Jurkat cells.

To date, only Bačkorová et al. [29] have evaluated Jurkat cell sensitivity to four common lichen secondary metabolites, parietin, atranorin, usnic acid, and gyrophoric acid. Their results showed concentration- and time-dependent cytotoxicity using the MTT assay, with IC_50_ values ranging from 76.3 µM to above 200 µM, usnic acid being the most potent. Moreover, they noted that lichen extracts exhibited greater cytotoxicity than individual compounds, likely due to synergistic or additive interactions among their components [12,28,30,31].

Several studies have evaluated the cytotoxicity of *Pseudevernia furfuracea* and lichens compounds in different cells lines [12,13,14,15,16,30,32,33,34]. In comparative assays evaluating nine human cancer cell lines exposed to parietin, atranorin, usnic acid, and gyrophoric acid, Jurkat cells exhibited one of the highest sensitivities [29]. The cytotoxicity showed by HE1 and HE2 in the present work was similar to the results reported by Kosanic et al. [12] in human melanoma (FemX) and LS 174 (human colon carcinoma) cell lines after exposure to lichen extracts and compounds of *Pseudevernia furfuracea* (12.5–200 µg/mL) by MTT test. The IC_50_ values were 55.09 and 56.96 µg/mL in the FemX and LS 174 cell lines, respectively. Other authors assessed the cytotoxicity of different *P. furfuracea* extracts (methanol, acetone, and ethyl acetate) in colon cancer cells (HTC-116 and SW-480) and normal human fibroblast cell line (MRC-5) by MTT test [32]. The highest cytotoxicity was observed after 72 h of exposure to ethyl acetate and acetone *P. furfuracea* extracts, with reported IC_50_ values of 21.2 µg/mL on HTC-116 and 51.3 µg/mL on SW-480 cells. The studied extracts produced considerable cytotoxicity on cancer cells, while non-cytotoxicity was observed on normal cells. Overall, the cytotoxic effects of lichens have been reported across multiple cancer cell lines, with their activity generally higher in carcinoma cells than in non-cancerous cells [35].

Ingelfinger et al. [33] studied the cytotoxic effects produced by *P. furfuracea* extracts (3 and 30 µg/mL) in HMEC-1 and HCT-116 cells using resazurin and WST-1 assays, respectively; after exposure, cell viability was not affected in both cell lines. The cytotoxic effects of different acetone extracts including *P. furfuracea* (12.5–200 µg/mL) in different cancer cell lines (22RV1, HT-29, Hep-G2, and CHO) were evaluated by WST1 assay, and *P. furfuracea* produced the highest effects in all cancer cell lines assessed, with IC_50_ values ranged from 42.30 µg/mL (22RV1 cells) to 68.60 µg/mL (Hep-G2 cells) [13]. Other authors did not observe cytotoxic effects in the HEK 293T cell line after exposure to methanol extract (PF70M) of *P. furfuracea* (0.156–100 µg/mL) using Alamar blue [14]. The same authors evaluated methyl haematommate (PF-1) and atraric acid (PF-2) (10–80 µg/mL), polyphenolic molecules isolated from *P. furfuracea*, in five cancer cell lines (MDA-MB-231, MCF7, PA-1, HepG2, and SCC-40) [34]. The compounds studied did not show significant antiproliferative affects against MDA-MB-231, HepG2, and SCC-40 cell lines; however, significant activity was observed in PA-1 cells after exposure to atraric acid and moderate activity in the case of MCF-7 cells. Petrova et al. [30] assessed the cytotoxicity of *P. furfuracea* extract (PSE) and physodic acid (10–100 µg/mL) in different cell lines (MCF-10A, Bj-5ta, and HUVECs) by MTS assay. These authors reported different sensitivities in cell lines after exposure to PSE and Phy, with IC_50_ values between 45.53 to 92.70 µg/mL for PSE and 79.88 to 92.76 µg/mL for Phy. However, the findings indicated that neither compound exhibited marked cytotoxicity in any of the tested cell lines. Kello et al. [15] assayed in nine cancer cell lines (Jurkat, HeLa, HTC116, Caco-2, MCF-7, MDA-MB-231, A549, BLM, and A2780) *P. furfuracea* extracts (10–100 µg/mL). In these 2D systems, *P. furfuracea* extract was one of the best sources of potential cytotoxic substances with IC_50_ values in the range of 31.9–48.6 µg/mL. Furmanek et al. [16] assessed the cytotoxic potential of different extracts from lichen species, including *P. furfuracea* (1–500 µg/mL) in five human cancer cell lines (Jurkat, MCF-7, Caco-2, SK-mel-28, and U87MG). The highest cytotoxic activity was shown by the *P. furfuracea* extract, which inhibited cell viability of MCF-7 (IC_50_: 110.84 µg/mL), Caco-2 (IC_50_: 123.86 µg/mL), SK-mel-28 (IC_50_: 161.80 µg/mL), U87MG (IC_50_: 107.43 µg/mL), and Jurkat (IC_50_: 174.87 µg/mL), higher values compared to those obtained in the present work.

Once the cytotoxicity of HE1 and HE2 was established, the results were used to investigate toxicity mechanisms in a tiered approach.

### 3.4. Analysis of Cell Death Mechanisms (Apoptosis/Necrosis) Through Flow Cytometry

**Figure 4:**
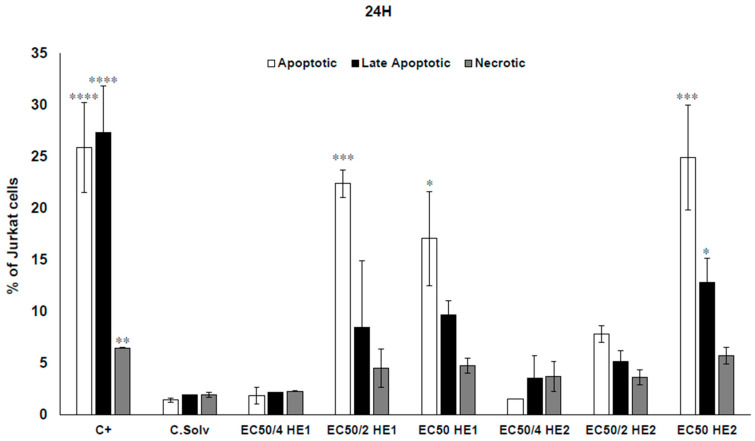
Jurkat cells were treated with HE1 or HE2 at EC_50_, EC_50/2_, and EC_50/4_ concentrations for 24 h. Apoptosis and necrosis were quantified by dual staining with Annexin V-FITC (early apoptosis) and Propidium Iodide (PI, necrosis marker). Cells were analyzed by flow cytometry (10,000 events/sample) using a MACSQuant VYB cytometer. Camptothecin (1.5 μM) served as positive control and 0.2% DMSO as solvent control. Values are mean ± SD of three replicates. Statistical significance: * *p* < 0.05, ** *p* < 0.01, *** *p* < 0.001, and **** *p* < 0.0001.

**Figure 5:**
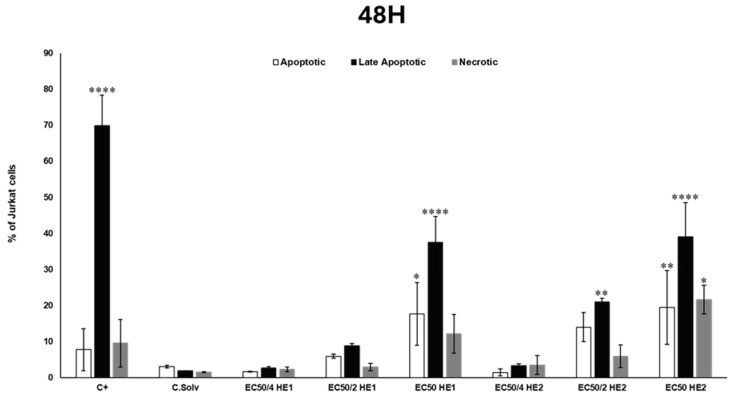
Jurkat cells were treated with HE1 or HE2 at EC_50_, EC_50/2_, and EC_50/4_ concentrations for 48 h. Apoptosis and necrosis were quantified by dual staining with Annexin V-FITC (early apoptosis) and Propidium Iodide (PI, necrosis marker). Cells were analyzed by flow cytometry (10,000 events/sample) using a MACSQuant VYB cytometer. Camptothecin (1.5 μM) served as positive control and 0.2% DMSO as solvent control. Values are mean ± SD of three replicates. Statistical significance: * *p* < 0.05, ** *p* < 0.01, and **** *p* < 0.0001.

The apoptotic/necrotic effects of HE1 and HE2 were assessed in Jurkat cells. The results obtained are shown in Figure 4 and Figure 5 for 24 and 48 h, respectively. The concentrations evaluated for both HE1 and HE2 were EC_50_, EC_50/2_, and EC_50/4_ in the case of HE1 (53.79, 26.89, and 13.45 µg/mL) and for HE2 (59.76, 29.88, and 14.94 µg/mL).

Regarding HE1 and HE2, more than 90% of viable cells were observed in the solvent control group (0.2% DMSO) for both exposure times. In the positive control group, between 60 and 90% of cells were apoptotic, late apoptotic, or necrotic after exposure for 24 or 48 h.

In the case of HE1, no significant differences were detected after 24 h exposure to 13.45 µg/mL (EC_50/4_). However, at the highest concentrations assayed (EC_50/2_ and EC_50_), significant differences in the percentage of apoptotic (22.4 ± 1.36 and 17.10 ± 4.55%, respectively) cells in comparison to the solvent control group were observed (Figure 4). After 48 h of exposure, no significant differences were observed at either 13.45 or 26.89 µg/mL of HE1. Nevertheless, the highest concentration tested exhibited a significant increase in both the apoptotic and late apoptotic populations, the late apoptotic being predominant with 37.54 ± 7.10% of cells (Figure 5).

With respect to HE2, after 24 h, no significant differences were observed in the 29.88 and 14.94 µg/mL groups. However, at the highest concentration evaluated, apoptosis and late apoptosis (24.93 ± 5.05% and 12.86 ± 2.34%, respectively) were the predominant death mechanisms (Figure 4). At 48 h, no significant differences were observed at the lowest concentration of HE2 (14.94 µg/mL). In the 29.88 µg/mL exposed group, significant differences in comparison to the solvent control group were detected only in the late apoptotic population (21.05 ± 1.00%). Finally, a significant increase in the percentage of apoptotic, late apoptotic, and necrotic populations was observed at EC_50_, with the most significant increase in the case of late apoptotic cells with 39.03 ± 9.53% of cells (Figure 5).

Compounds produced by lichens exert cytotoxic effects by inducing cell cycle arrest, apoptosis, necrosis, and inhibition of angiogenesis [3,36]. To our knowledge, this is the first study that evaluates the mechanisms of cell death (apoptosis/necrosis) produced by these novel types of extracts from *P. furfuracea* (HE1 and HE2) in Jurkat cells. Seklić et al. [32] reported that apoptotic responses are influenced by treatment dose and exposure time, with stronger effects arising after extended incubation periods, which aligns with the results obtained in this work: more predominancy of late apoptotic cells at 48 h (vs. 24 h) for both extracts and even necrotic cells at the higher concentration for HE2.

As far as we know, only Kello et al. [28] assayed the ability of a *P. furfuracea* extract (45 µg/mL) to produce cell death (apoptosis) in Jurkat cells. The authors reported alterations in cell cycle dynamics, noting S-phase arrest after 24 h of exposure and a progressive accumulation of cells in the sub-G_0_/G_1_ phase, accompanied by a decrease in the G_1_ and G_2_/M populations between 24 and 72 h. These findings indicate that the lichen extract triggered apoptosis-mediated cell death in Jurkat cells, consistent with the results of the present study.

Other authors have studied the ability of *P. furfuracea* extracts to cause cell death in different cell lines [12,30,32,33]. Thus, methanol, acetone, and ethyl acetate extracts of *P. furfuracea* (10 and 100 µg/mL) caused cell death mainly by apoptosis in HCT-116 and SW-480 cells [32]. The authors reported that the ethyl acetate extract exhibited strong pro-apoptotic activity in HCT-116 cells after 72 h of exposure, inducing 20.12% late apoptosis at the lowest concentration and 75.39% late apoptotic and 5.62% necrotic cells at the highest dose. In SW-480 cells, the methanol and acetone extracts produced the most pronounced pro-apoptotic effects, consistent with their observed cytotoxicity profiles. Overall, the cytotoxicity of *P. furfuracea* extracts in HCT-116 and SW-480 cells were the consequence of induced apoptosis. In contrast, Ingelfinger et al. [33] reported no evidence of apoptosis or cell cycle phase alterations in HCT-116 cells following exposure to 3 and 30 µg mL^−1^ of the extract.

Several well-known lichen secondary metabolites have also been investigated for their capacity to induce cell death. [29,37]. Among the tested metabolites (parietin, atranorin, usnic acid, and gyrophoric acid), all except parietin induced alterations in cell cycle distribution across four cancer cell lines (A2780, HCT-116 p53^+/+^, HCT-116 p53^−/−^, and HL-60). Atranorin produced effects only in HL-60 cells at 100 µM after 72 h; by contrast, no effect was observed in this cell line after usnic acid exposure. In addition, gyrophoric acid was only effective in A2780 cells. The authors concluded that alterations in cell cycle progression could be attributed to the biological action of the tested metabolites. Moreover, usnic acid and atranorin induced apoptosis in HT-29 and A2780 cancer cells, being activators of programmed cell death in both cell lines [37]. Lichen-derived compounds act as modulators of apoptosis in diverse cancer cells [38,39], primarily through the regulation of genes such as caspases, p53, p38, and Bcl-2 family members, which govern cellular survival and apoptotic processes [40].

Various lichen-derived compounds have been shown to induce apoptosis through multiple mechanisms, including mitochondrial pathways, death receptor signaling, cellular signaling cascades, and hypoxia-related responses [37,41]. Regarding the mechanism of apoptosis, activation of death receptors leads to the initiation of caspases, which are essential proteases responsible for executing programmed cell death. The ability of certain lichen metabolites to trigger apoptosis underscores their potential as pro-apoptotic agents for the targeted elimination of tumor cells [42]. Apoptosis induced by these metabolites may proceed via the intrinsic mitochondrial route or the extrinsic death receptor–mediated pathway, both involving specific and independent protein cascades [32]. Within the extrinsic apoptotic pathway, cell death is triggered by the interaction of pro-apoptotic ligands (e.g., TNF-α) with membrane death receptors such as TNFR1/TNFRSF1A [42]. Further investigations are warranted to elucidate the specific molecular pathways through which lichen extracts and their bioactive components induce apoptosis.

### 3.5. Effects of HE1 and HE2 on Cytokine mRNA Expression by RT-qPCR

**Figure 6:**
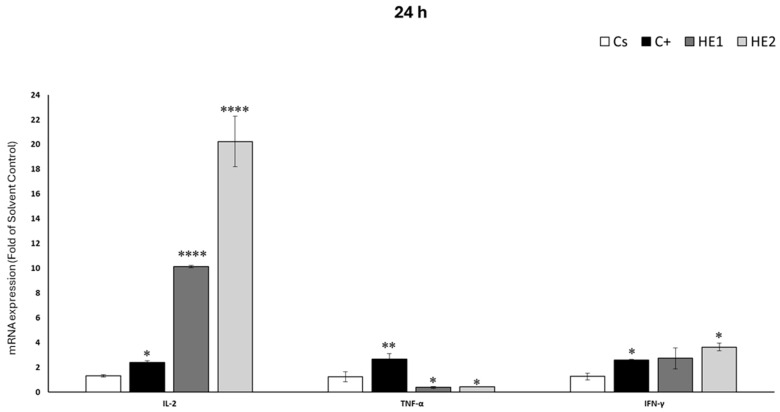
Jurkat cells were exposed for 24 h to non-cytotoxic concentrations of HE1 or HE2 (20 μg/mL). Relative mRNA expression of IL-2, TNF-α, and IFN-γ was quantified by RT-qPCR using GAPDH as housekeeping gene. Lipopolysaccharide (LPS, 10 ng/mL) served as positive control and 0.2% DMSO as solvent control. Expression levels are represented as fold change (2^−ΔΔCT^) relative to solvent control. Results are mean ± SD of three independent experiments. Significance: * *p* < 0.05, ** *p* < 0.01, and **** *p* < 0.0001.

**Figure 7:**
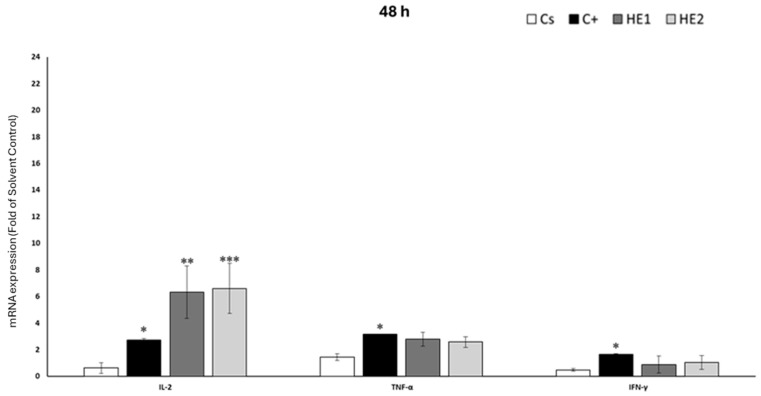
Jurkat cells were exposed for 48 h to non-cytotoxic concentrations of HE1 or HE2 (20 μg/mL). Relative mRNA expression of IL-2, TNF-α, and IFN-γ was quantified by RT-qPCR using GAPDH as housekeeping gene. Lipopolysaccharide (LPS, 10 ng/mL) served as positive control and 0.2% DMSO as solvent control. Expression levels are represented as fold change (2^−ΔΔCT^) relative to solvent control. Results are mean ± SD of three independent experiments. Significance: * *p* < 0.05, ** *p* < 0.01, and *** *p* < 0.001.

Cytokine gene expression profiles are shown in Figure 6 and Figure 7, illustrating responses after 24 h and 48 h of treatment, respectively. In the case of HE1, IFN-γ expression after 24 h of exposure did not differ significantly from that of the solvent control group. Conversely, treatment with HE1 resulted in a marked increase in IL-2 expression (10.14-fold) and a significant decrease in TNF-α levels (0.64-fold compared with the solvent control) (Figure 6). After 48 h (Figure 7), no significant differences were observed in TNF-α and IFN-γ, with values close to those of the solvent control group. For HE2, only IL-2 expression was significantly upregulated, showing a 6.32-fold increase. For HE2, exposure for 24 h resulted in a marked increase in the expression of IL-2 (20.24-fold) and IFN-γ (3.62-fold) relative to the solvent control (Figure 6). On the contrary, down-regulation was observed in TNF-α (0.59-fold with respect to the solvent control). After 48 h, no changes were found for TNF-α and IFN-γ expression. At 48 h of exposure, a significant up-regulation of IL-2 was detected, corresponding to a 6.59-fold increase relative to the solvent control (Figure 7).

This study is the first to report that *Pseudevernia furfuracea* extracts modulate mRNA expression levels of key cytokines (IL-2, TNF-α, and IFN-γ) in Jurkat cells. The observed up-regulation of IL-2 after 24 and 48 h of exposure to the extracts suggests a potential immunomodulatory effect, as IL-2 plays a central role in immune activation and homeostasis [43]. This cytokine regulates the magnitude and duration of both primary and memory immune responses, promotes T-cell proliferation, enhances the cytotoxic activity of natural killer (NK) cells, and induces the release of pro-inflammatory mediators [44]. TNF-α is a multifunctional cytokine involved in immune regulation, apoptosis, and cell differentiation [45]. It showed only a modest response: a significant decrease was observed at 24 h, but no change was detected at 48 h. Given TNF-α’s dual role in inducing both apoptosis and necrosis depending on the cellular context [46], this mild modulation suggests a limited effect in comparison to IL-2. IFN-γ expression increased only in response to the HE2 extract at 24 h, with no changes observed at 48 h. This pattern may reflect an early-stage adaptive immune response but with less sustained activation compared to IL-2. Overall, these findings suggest that *P. furfuracea* extracts, particularly HE2, may influence immune responses through selective and time-dependent modulation of cytokine gene expression.

**Figure 8:**
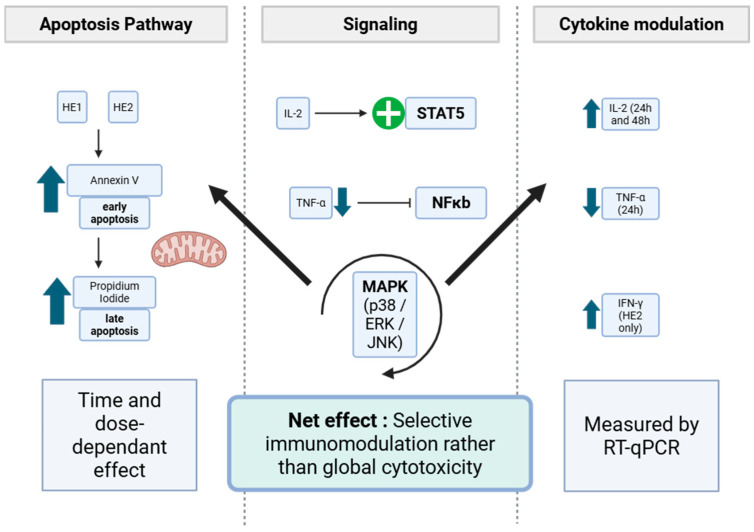
Proposed immunomodulatory mechanisms of *Pseudevernia furfuracea* hydrodistillation-derived extracts (HE1 and HE2) in Jurkat cells. Flow cytometry results (Annexin V/PI staining) show a concentration and time-dependent progression from apoptotis to late apoptosis. RT-qPCR analyses reveal modifications (up-regulation and/or down-regulation) in chemokine expression (IL-2, TNF-α, and IFN-γ). The proposed signaling interactions include activation of STAT5 by IL-2, modulation of NFκB associated with TNF-α suppression, and involvement of MAPK (ERK, JNK, p38) cascades linking cytokine regulation to apoptosis. In the diagram, thick blue arrows indicate up- or down-regulation; the green ‘+’ sign indicates activation, and the T-bar indicates inhibition.

The observed cytokine modulation suggests that *P. furfuracea* extracts may influence key intracellular signaling pathways governing T-cell activation and apoptosis. The up-regulation of IL-2 and IFN-γ, together with the reduction of TNF-α, indicates possible activation of STAT5 and inhibition of NF-κB pathways [47,48]. Previous studies have demonstrated that lichen metabolites, such as physodic acid, can modulate MAPK signaling pathways (ERK, JNK, and p38) and induce apoptosis [28]. Taken together, these findings support the involvement of STAT5, NF-κB, and MAPK signaling in the immunomodulatory response observed [49,50]. A schematic representation summarizing the proposed mechanisms connecting apoptosis pathway and chemokine expression is presented in Figure 8.

To date, only one study has examined the effects of a *Pseudevernia furfuracea* methanolic extract (10 and 50 µg mL^−1^) on the expression of anti-migratory/pro-migratory and invasion-related markers (*E-cadherin*, *β-catenin*, *N-cadherin*, *Vimentin*, *Snail*, and *MMP-9*) in HCT-116 and SW-480 cells using qRT-PCR [51]. In HCT-116 cells, treatment increased *E-cadherin* expression while reducing *β-catenin*, *N-cadherin*, *Vimentin*, *Snail*, and *MMP-9* levels. In SW-480 cells, *P. furfuracea* extract suppressed all pro-migratory and invasive gene expressions. These findings indicate that the methanolic extract of *P. furfuracea* significantly modulates the expression of genes implicated in cell migration and invasion in both HCT-116 and SW-480 cell lines.

While these findings reveal the significant immunomodulatory and pro-apoptotic effects of *Pseudevernia furfuracea* hydrodistillation-derived extracts in Jurkat cells, the results are limited to an in vitro T-cell model. To establish their physiological and pharmacological relevance, future work should include in vivo studies or assays using primary human lymphocytes or animal models. Such investigations will be essential to confirm the systemic immunomodulatory capacity, bioavailability, and safety of the active compounds identified in this study.

## 4. Conclusions

The *Pseudevernia furfuracea* extracts HE1 and HE2 were analyzed by UHPLC-MS/MS for the first time, revealing several novel components, including Caprolactam, N,N-Diethylaniline, Erucamide, and 4-Isopropylaniline. Additionally, potential metabolites of atraric acid, present in both extracts, were characterized. HE1 and HE2 exhibited immunomodulatory effects on human Jurkat cells, as evidenced by cytotoxic activity primarily mediated through apoptosis after 24 h and progressing to late apoptosis at 48 h. Moreover, the expression of pro-inflammatory genes was modulated, with each extract eliciting a distinct expression pattern. Further studies are needed to elucidate the molecular mechanisms driving the immunomodulatory activity and to pinpoint the bioactive constituents accountable for the observed responses.

## Figures and Tables

**Table 1:** Putative identification of the compounds detected in HE1 and HE2 through UHPLC-MS/MS. Signal colors ranges vary from red representing low intensity, to yellow, indicating high intensity.

Name	Molecular Formula	Molecular Weight	RT	Area Max	HE1	HE2
(-)-Menthylacetate	C_12_H_22_O_2_	198.16156	16.606	5.33 × 10^6^	-	+
[FAhydroxy(20:3)]11_12-dihydroxy-5Z_8Z_14Z-eicosatrienoicacid	C_20_H_34_O_4_	338.24515	15.138	2.65 × 10^7^	-	+
1,2,2,6,6-Pentamethyl-4-piperidinyl acrylate	C_13H23_NO_2_	225.17225	17.992	1.64 × 10^7^	+	+
1_3_8-Naphthalenetriol	C_10_H_8_O_3_	176.04704	10.793	1.48 × 10^7^	-	+
2,4-Diethyl-thioxanthen-9-one	C_17_H_16_OS	268.09157	17.101	4.37 × 10^6^	+	+
2,6-Diethylaniline	C_10_H_15_N	149.11994	0.595	4.59 × 10^6^	+	+
2-oxa-4-azatetracyclo [6.3.1.1~6,10~.0~1,5~]tridecan-3-one	C_11_H_15_NO_2_	193.11005	0.562	1.29 × 10^7^	+	+
3,5-di-tert-Butyl-4-hydroxybenzaldehyde	C_15_H_22_O_2_	234.16151	14.297	8.06 × 10^6^	-	+
4-Isopropylaniline	C_9_H_13_N	135.1044	0.571	1.98 × 10^7^	+	+
4-O-acetyl hygrophorone A14	C_22_H_38_O_5_	382.27127	15.169	3.36 × 10^7^	-	+
4-Pentylaniline	C_11_H_17_N	163.13567	18.945	1.01 × 10^7^	+	-
5-(6-hydroxy-6-methyloctyl)-2,5-dihydrofuran-2-one	C_13_H_22_O_3_	243.18278	18.098	9.90 × 10^6^	+	+
9,10-phenanthraquinone	C_14_H_8_O_2_	208.05211	11.148	2.70 × 10^7^	-	+
Acetone oxime	C_3_H_7_NO	73.05259	0.595	1.27 × 10^7^	+	+
Bacillamidin G	C_17_H_35_NO	269.27114	17.66	3.67 × 10^6^	+	+
Berkazaphilone A	C_13_H_16_O_3_	220.10947	14.611	6.35 × 10^6^	-	+
Caprolactam	C_6_H_11_NO	113.08384	2.766	2.62 × 10^8^	-	+
Cladoacetal B	C_12_H_12_O_3_	204.07826	15.576	6.66 × 10^6^	-	+
DEET	C_12_H_17_NO	191.13064	9.503	6.24 × 10^6^	-	+
Diaminotoluene	C_7_H_10_N_2_	122.08408	0.568	3.84 × 10^6^	+	-
Dibutyl hexanedioate	C_14_H_26_O_4_	258.18266	15.236	1.36 × 10^7^	-	+
Dibutyl phthalate	C_16_H_22_O_4_	278.15151	15.452	8.12 × 10^7^	+	+
Diethyl phthalate	C_12_H_14_O_4_	222.08883	10.794	1.02 × 10^7^	-	+
diisopropylethylamine	C_8_H_19_N	129.15148	18.346	7.73 × 10^7^	-	+
Di-N-butylnitrosoamine	C_8_H_18_N_2_O	158.14157	2.884	9.59 × 10^6^	-	+
Erucamide	C_22_H_43_NO	337.33387	19.148	1.63 × 10^8^	+	+
Eugenol methyl ether	C_11_H_14_O_2_	178.09907	6.501	6.67 × 10^6^	-	+
F1839-B	C_24_H_33_NO_6_	431.23015	11.211	1.59 × 10^7^	+	+
FL6DDAGI0001_a	C_22_H_26_O_6_	386.1723	11.212	2.25 × 10^7^	+	+
Heptaminol	C_8_H_19_NO	145.14635	0.569	2.33 × 10^6^	+	+
MDPBP	C_15_H_19_NO_3_	261.13602	15.027	5.62 × 10^6^	-	+
N,N-Diethylaniline	C_10_H_15_N	149.11997	19.072	1.88 × 10^8^	+	+
N-Methylpyridinium	C_6_H_7_N	93.05765	0.562	1.43 × 10^6^	+	-
NP-014924	C_10_H_12_O_4_	164.04719	9.917	5.75 × 10^7^	+	+
NP-020014	C_15_H_26_O_3_	276.17208	15.028	1.31 × 10^7^	-	+
Oleamide	C_18_H_35_NO	281.2712	17.717	3.72 × 10^6^	+	-
Phenmetrazine	C_11_H_15_NO	177.11504	0.563	6.46 × 10^6^	+	+
Phthalic anhydride	C_8_H_4_O_3_	148.0158	10.793	1.50 × 10^7^	-	+
Pseudocapsaicin	C_17_H_27_NO_3_	293.19865	14.61	1.54 × 10^7^	-	+
Stearoyl ethanolamide	C_20_H_41_NO_2_	309.30227	18.438	1.40 × 10^7^	+	+
Thermolide E	C_28_H_53_NO_8_	531.37608	15.177	1.36 × 10^7^	-	+
Tributyl phosphate	C_12_H_27_O_4_P	266.16432	14.018	6.15 × 10^6^	-	+
Triethylene glycol	C_6_H_14_O_4_	150.08891	0.572	1.38 × 10^7^	-	+

**Table 2:** Putative identification of atraric acid transformation products by UHPLC-MS/MS. Signal color ranges vary from red, representing low intensity, to green, indicating high intensity.

Transformation	Composition Change	Molecular Formula	Molecular Weight	RT	Area Max	HE1	HE2
Dehydration, Desaturation	-(H_4_O)	C_10_H_8_O_3_	176.04705	10.794	1.45 × 10^7^	+	+
Nitro Reduction, Oxidation	-(CO)	C_9_H_12_O_3_	168.07819	14.915	1.96 × 10^6^	+	-
Nitro Reduction, Oxidation	-(CO)	C_9_H_12_O_3_	168.07816	18.579	1.89 × 10^6^	-	+
Dehydration	-(CH_4_O)	C_9_H_8_O_3_	164.04704	9.918	5.87 × 10^6^	+	+
Nitro Reduction, Oxidation	-(CO)	C_9_H_12_O_3_	168.07816	17.633	1.56 × 10^6^	-	+
Nitro Reduction, Oxidation	-(CO)	C_9_H_12_O_3_	168.07823	15.827	2.55 × 10^6^	+	-
Nitro Reduction, Oxidation	-(CO)	C_9_H_12_O_3_	168.07817	16.709	1.51 × 10^6^	+	-
Nitro Reduction, Oxidation	-(CO)	C_9_H_12_O_3_	168.07823	16.169	1.40 × 10^6^	+	-
Nitro Reduction, Oxidation	-(CO)	C_9_H_12_O_3_	168.0782	16.867	1.39 × 10^6^	+	+
Nitro Reduction, Oxidation	-(CO)	C_9_H_12_O_3_	168.07823	16.015	3.33 × 10^6^	+	-
Dehydration, Reduction, Acetylation	+(C_2_H_2_)	C_12_H_14_O_4_	222.08885	10.793	1.03 × 10^7^	-	+
Nitro Reduction, Oxidation	-(CO)	C_9_H_12_O_3_	168.0782	19.35	1.18 × 10^6^	+	-
Nitro Reduction, Oxidation	-(O) + (H_2_)	C_10_H_14_O_3_	182.09375	18.056	6.99 × 10^5^	+	-
Nitro Reduction, Methylation	-(O_2_) + (CH_4_)	C_11_H_16_O_2_	180.11452	18.726	3.98 × 10^5^	+	-
Nitro Reduction, Oxidation	-(CO)	C_9_H_12_O_3_	168.0782	16.625	9.97 × 10^5^	+	-
Nitro Reduction, Oxidation	-(CO)	C_9_H_12_O_3_	168.0782	16.928	9.96 × 10^5^	+	+
Nitro Reduction, Oxidation	-(CO)	C_9_H_12_O_3_	168.07819	16.204	8.93 × 10^5^	+	-
Nitro Reduction, Oxidation	-(CO)	C_9_H_12_O_3_	168.07816	17.52	2.93 × 10^5^	+	-
Nitro Reduction, Oxidation	-(CO)	C_9_H_12_O_3_	168.0782	16.248	7.91 × 10^5^	+	-
Nitro Reduction, Methylation	-(O_2_) + (CH_4_)	C_11_H_16_O_2_	180.1145	17.584	3.91 × 10^5^	-	+
Nitro Reduction, Methylation	-(O_2_) + (CH_4_)	C_11_H_16_O_2_	180.11461	18.295	3.91 × 10^5^	-	+
Nitro Reduction, Oxidation	-(CO)	C_9_H_12_O_3_	168.0782	16.882	1.09 × 10^6^	-	+
Nitro Reduction, Methylation	-(O_2_) + (CH_4_)	C_11_H_16_O_2_	180.11449	17.825	3.80 × 10^5^	+	-
Nitro Reduction, Oxidation	-(CO)	C_9_H_12_O_3_	168.07817	18.63	5.77 × 10^5^	-	+
Nitro Reduction, Oxidation	-(CO)	C_9_H_12_O_3_	168.07814	18.686	7.76 × 10^5^	+	-
Nitro Reduction, Methylation	-(O_2_) + (CH_4_)	C_11_H_16_O_2_	180.11453	18.576	3.76 × 10^5^	+	-
Nitro Reduction, Oxidation	-(CO)	C_9_H_12_O_3_	168.07817	17.483	3.76 × 10^5^	-	+
Nitro Reduction, Methylation	-(O_2_) + (CH_4_)	C_11_H_16_O_2_	180.1145	18.195	3.75 × 10^5^	+	-
Nitro Reduction, Oxidation	-(CO)	C_9_H_12_O_3_	168.07818	18.86	9.74 × 10^5^	+	+
Nitro Reduction, Methylation	-(O_2_) + (CH_4_)	C_11_H_16_O_2_	180.11453	18.628	3.74 × 10^5^	+	-
Nitro Reduction, Oxidation	-(CO)	C_9_H_12_O_3_	168.07816	18.305	7.73 × 10^5^	+	+
Nitro Reduction, Reduction	-(CO_2_) + (H_2_)	C_9_H_14_O_2_	154.09894	16.789	4.72 × 10^5^	+	-
Nitro Reduction, Oxidation	-(CO)	C_9_H_12_O_3_	168.07818	19.284	9.69 × 10^5^	+	-
Desaturation, Nitro Reduction, Methylation	-(O_2_) + (CH_2_)	C_11_H_14_O_2_	178.09886	17.621	4.64 × 10^5^	+	-
Nitro Reduction, Reduction	-(CO_2_) + (H_2_)	C_9_H_14_O_2_	154.09892	16.424	4.54 × 10^5^	-	+
Nitro Reduction, Oxidation	-(CO)	C_9_H_12_O_3_	168.0782	17.32	1.05 × 10^6^	-	+
Nitro Reduction, Oxidation	-(CO)	C_9_H_12_O_3_	168.07823	17.076	9.48 × 10^5^	+	-
Nitro Reduction, Methylation	-(O_2_) + (CH_4_)	C_11_H_16_O_2_	180.1145	17.51	5.47 × 10^5^	-	+
Nitro Reduction, Oxidation	-(CO)	C_9_H_12_O_3_	168.07819	17.491	1.04 × 10^6^	+	-
Desaturation, Nitro Reduction, Methylation	-(O_2_) + (CH_2_)	C_11_H_14_O_2_	178.09885	18.906	3.35 × 10^5^	+	+
Nitro Reduction, Reduction	-(CO_2_) + (H_2_)	C_9_H_14_O_2_	154.09893	16.21	4.30 × 10^5^	+	+
Nitro Reduction, Oxidation	-(CO)	C_9_H_12_O_3_	168.07814	17.505	3.30 × 10^5^	-	+
Desaturation, Nitro Reduction	-(O_2_)	C_10_H_12_O_2_	164.08324	18.52	3.24 × 10^5^	-	+
Nitro Reduction, Oxidation	-(CO)	C_9_H_12_O_3_	168.07817	17.191	3.23 × 10^5^	+	-
Nitro Reduction, Reduction, Acetylation	-(O) + (C_2_H_6_)	C_12_H_18_O_3_	210.12503	18.342	3.19 × 10^5^	-	+
Desaturation, Nitro Reduction, Methylation	-(O_2_) + (CH_2_)	C_11_H_14_O_2_	178.09889	18.742	4.11 × 10^5^	+	-
Desaturation, Nitro Reduction, Methylation	-(O_2_) + (CH_2_)	C_11_H_14_O_2_	178.09885	17.504	5.07 × 10^5^	-	+
Nitro Reduction, Oxidation	-(CO)	C_9_H_12_O_3_	168.07816	18.906	7.03 × 10^5^	-	+
Nitro Reduction	-(CO_2_)	C_9_H_12_O_2_	152.08328	16.93	5.03 × 10^5^	-	+
Nitro Reduction, Reduction	-(CO_2_) + (H_2_)	C_9_H_14_O_2_	154.09892	16.599	6.02 × 10^5^	+	-

## Data Availability

The original contributions presented in this study are included in the article material. Further inquiries can be directed to the corresponding author.

## Abbreviations

The following abbreviations are used in this manuscript:

UHPLC-MS/MS	ultra-high-performance liquid chromatography coupled with tandem mass spectrometry
RT-qPCR	quantitative real-time polymerase chain reaction
IL-2	interleukin 2
TNF-α	tumor necrosis factor α
IFN-γ	interferon γ
EC_50_	effective concentration 50
2D	two-dimensional
3D	three-dimensional
mRNA	messenger ribonucleic acid
C-	negative reaction to calcium hypochlorite
K-	negative reaction to potassium hydroxide
K+	positive reaction to potassium hydroxide
GC-MS	Gas chromatography coupled to mass spectrometry
ATCC	American Type Culture Collection
DMSO	dimethylsulfoxyde
BB	annexin-binding buffer
Annexin V-FITC	annexin V conjugated with fluorescein isothiocyanate
GAPDH	glyceraldehyde-3-phosphate dehydrogenase
LPS	lipopolysaccharide
RT	reverse transcription
cDNA	complementary DNA
ANOVA	analysis of variance
NCI	American National Cancer Institute
IC50	inhibitory concentration 50
MTS assay	3-(4,5-dimethylthiazol-2-yl)-5-(3-carboxymethoxyphenyl)-2-(4-sulfophenyl)-2H-tetrazolium) assay
MTT test	3-(4,5-dimethylthiazol-2-yl)-2,5-diphenyltetrazolium bromide assay
WST-1 assay	water-soluble tetrazolium-1 assay
phy	physodic acid
